# Systematic reviews of empirical literature on bioethical topics: Results from a meta-review

**DOI:** 10.1177/0969733020907935

**Published:** 2020-04-02

**Authors:** Marcel Mertz, Hélène Nobile, Hannes Kahrass

**Affiliations:** Hannover Medical School, Germany

**Keywords:** Bioethics, empirical ethics, empirical literature, knowledge syntheses, systematic review

## Abstract

**Background:**

In bioethics, especially nursing ethics, systematic reviews are increasingly popular. The overall aim of a systematic review is to provide an overview of the published discussions on a specific topic. While a meta-review on systematic reviews on normative bioethical literature has already been performed, there is no equivalent for systematic reviews of empirical literature on ethical topics.

**Objective:**

This meta-review aims to present the general trends and characteristics of systematic reviews of empirical bioethical literature and to evaluate their reporting quality.

**Research design:**

Literature search was performed on PubMed and Google Scholar. Qualitative content analysis and quantitative approaches were used to evaluate the systematic reviews. Characteristics of systematic reviews were extracted and quantitatively analyzed. The reporting quality was measured using an adapted PRISMA checklist.

**Findings:**

Seventy-six reviews were selected for analysis. Most reviews came from the field of nursing (next to bioethics and medicine). Selected systematic reviews investigated issues related to clinical ethics (50%), followed by research ethics (36%) and public health ethics or organizational ethics (14%). In all, 72% of the systematic reviews included authors’ ethical reflections on the findings and 59% provided ethical recommendations. Despite the heterogeneous reporting of the reviews, reviews using PRISMA tended to score better regarding reporting quality.

**Discussion:**

The heterogeneity currently observed is due both to the interdisciplinary nature of nursing ethics and bioethics, and to the emerging nature of systematic review methods in these fields. These results confirm the findings of our previous review of systematic reviews on normative literature, thereby highlighting a recurring methodological gap in systematic reviews of bioethical literature. This also indicates the need to develop more robust methodological standards.

**Conclusion:**

Through its extensive overview of the characteristics of systematic reviews of empirical literature on ethical topics, this meta-review is expected to inform further discussions on minimal standards and reporting guidelines.

## Introduction

The overall aim of systematic reviews is to provide readers with an unbiased overview on specific topics discussed in the literature.^1,2^ In the interdisciplinary field of bioethics, systematic reviews can synthesize normative or empirical literature as well as a mix of both. On one hand, systematic reviews of normative literature provide an overview of ethical issues, arguments, reasons, values, or norms surrounding ethical topics, and are mostly, though not exclusively, drawn from philosophical or generally conceptual articles.^3^ On the other hand, systematic reviews of empirical literature aim at taking stock of data such as attitudes, preferences, opinions, experiences, and decision-making processes regarding the topics at hand, summarizing quantitative or qualitative social science studies.

Recent years have seen an increase in the number of published systematic or semi-systematic reviews in the field of bioethics.^4^ Since nursing involves human interactions in, often difficult, care situations, ethical issues are bound to routinely arise in nursing practice and it is therefore understandable that systematic reviews on nursing ethics are increasingly popular. In an earlier meta-review on reviews of normative literature,^4^ 15% (n = 17) were published in journals belonging to nursing, most frequently in *Nursing Ethics* (n = 7) and the *Journal of Advanced Nursing* (n = 4). Such reviews are gaining importance as they are not only addressed to ethics researchers but also to health professionals who can use them as knowledge syntheses on given ethical topics. On one hand, when they show a discrepancy between healthcare workers’ attitudes or preferences and daily routine, systematic reviews can help the identification of ethical issues. On the other hand, systematic reviews can provide crucial empirical evidence to support ethical reflections on healthcare practice. They are of further interest for the description of ethical aspects of Health Technology Assessment and for the development of ethical guidelines.^5,6^

Still, in bioethics, the use of this particular method of searching and synthesizing published information is relatively new when compared to other established fields such as medicine, public health, health technology assessment, or psychology. This is not only indicated by the small number of published reviews but also by the fact that its methodology is currently largely borrowed from the established fields. Methodological strategies such as choice of methods, application, reporting, and standards of quality for reporting have yet to be adapted for the specific field of bioethics. Such adaptation of existing methodological tools would need to include reflections on adequate search strategies (as, for example, “PICO” (population–intervention–comparison–outcome) is seldom useful); on the relation to normative-ethical concepts, norms, or values; on the analysis method for information units as diverse as patients’ opinions or evaluation of ethics tools; and on the discussion of the particular ethical relevance and/or implications of the findings (“ethical outcome”).

About a decade ago, Strech et al.^7^ started discussing the standards guiding the search, analysis, and synthesis strategies used in systematic reviews of empirical bioethical literature. Regarding systematic reviews of normative literature, our earlier publications reported some methodological shortcomings.^4,8^ In order to adapt such a methodology, it is essential to first gain an overview of the standards currently used in systematic reviews of bioethical literature, including the empirical one which has not been investigated yet.

## Objective

As an intermediary step toward the goal of developing such methodology, our current study aims at reviewing selected methodological features of systematic reviews on empirical literature on bioethical topics, as well as the reporting quality of the findings. In order to map the field of systematic reviews of empirical bioethical literature, we further documented review characteristics such as year of publication or academic affiliations of corresponding authors. Thus, in a certain way, this study is a further follow-up of earlier publications where we presented the results of systematic reviews of normative literature, which is why the structure was kept approximatively the same.^4,8^

## Research design

### Search/selection

**Figure 1.**
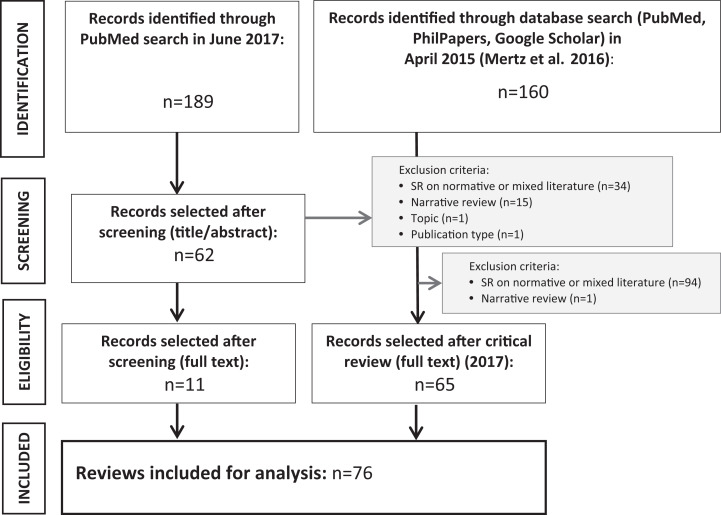
Selection process of reviews of empirical literature on bioethical topics.

The initial search (April 2015) for systematic reviews on bioethical topics used two PubMed searches, supported by searches in PhilPapers and Google Scholar. In total, 1393 hits were produced, and 160 finally included after title/abstract and full-text screening (only publications in English, German, or French were included). A detailed account of the original search and selection strategy can be found in Supplemental File S2 and in Mertz et al. 2016.^4^ Seventy-six hits were classified as reviews of empirical literature and therefore included in the present study; the other reviews were analyzed separately.^4,8^ At a later critical review of the results for the analysis, 11 hits were excluded. In an update of the search in PubMed using the same search strategy (June 2017), another 62 hits were retrieved for further inspection, of which 11 were selected for our current research. Seventy-six articles were thus included for the in-depth analysis (see Figure 1).

### Analysis

For the analysis, methods of qualitative content analysis were used.^9^ A detailed account of the used methods for analysis can be found in Mertz et al. 2016^4^ and specifically in Mertz et al. 2017.^8^ Relevant text sections were identified, retrieved, if needed paraphrased or summarized, and then subsumed in main- and subcategories of an adapted version of the original coding matrix from Mertz et al. 2016.^4^ The adapted coding matrix entailed both closed entries (i.e. yes/no, numbers, countries) and open entries (i.e. excerpts from original text, paraphrases, or summaries). All reviews were analyzed by Hélène Nobile and, to increase reliability of the analysis, the first 36 of them (46%) were independently double-coded by the other authors (Hannes Kahrass and Marcel Mertz). Observed agreement for the coding of closed answer modes was between 75% and 100% (average: 90%; Cohen’s Kappa average: 0.79). Disagreements were discussed to reach consensus among the three authors.

As the goal of the meta-review was to describe the way reviews on bioethical topics are conducted, we did not aim at assessing the overall quality of the included reviews. Instead, we assessed the reporting quality (see below) as part of the scope of this meta-review, and not as a criterion to exclude reviews or to assess the validity of the results of single reviews.

### Synthesis

Descriptive statistics were applied for the closed answer modes. Entries in subcategories with open answer modes were summarized, sorted according to overarching themes or categories, and then counted. Some of the results were compared with items of the PRISMA guideline^10^ in order to evaluate the reporting (see later Figure 3).

**Figure 2.**
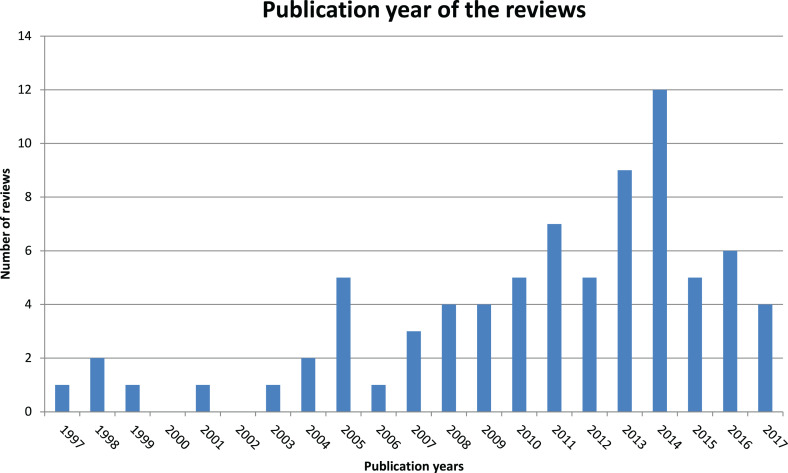
Publication years of the reviews.

**Figure 3.**
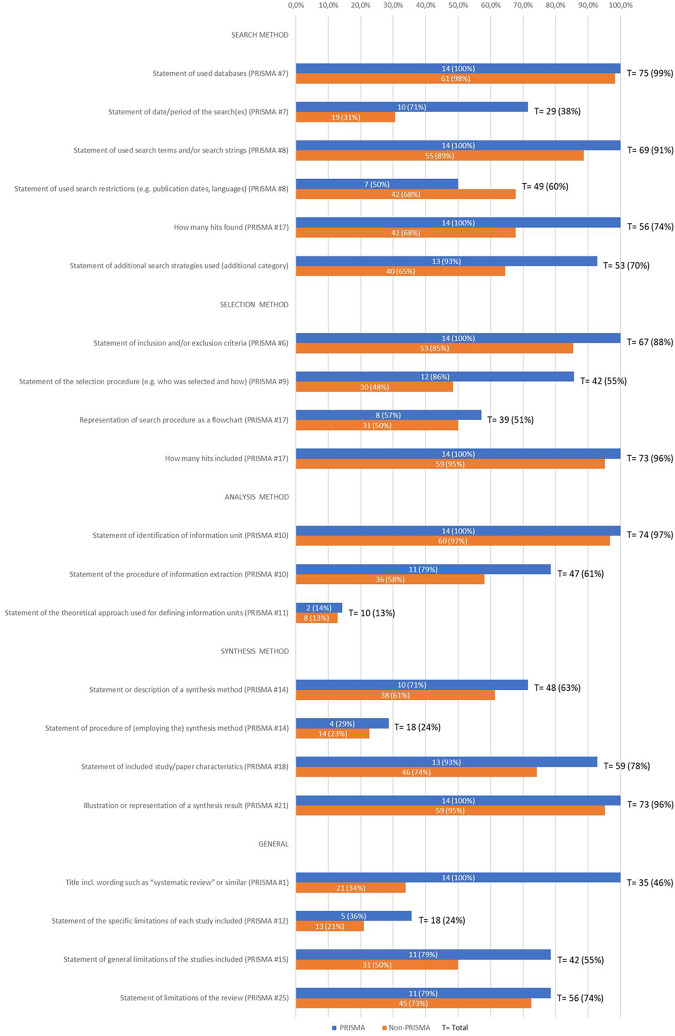
Reporting criteria fulfillment of the reviews: details (n = 76).

## Findings

Seventy-six reviews of empirical literature were included in the analysis (the full list of all included reviews, including bibliographic details, can be found in Supplemental File S1). In detail, 10 (13%) reviewed qualitative studies, 24 (32%) reviewed quantitative studies, and 42 (55%) were reviews of quantitative as well as qualitative studies.

### General characteristics

#### Languages, publication dates, and self-labeling

All reviews included were in English and published between 1997 and 2017. Sixty-three reviews (83%) were published in the last 10 years (2007–2017) (see Figure 2). In their titles, 35 (46%) labelled themselves as “systematic review,” including variants such as “systematic literature review,” “systematic qualitative review,” “systematic review of qualitative evidence,” or “mixed-method systematic review.” 17 (22%) used “literature review” including “structured literature review,” 3 (4%) used the term “review.” The following formulations came up only once (1%): “thematic synthesis of qualitative studies,” “meta-analysis,” “cross-cultural comparative review,” “critical review of the literature,” “synthesis of qualitative evidence,” “meta-synthesis of qualitative research,” and “bibliometric analysis.” The remaining reviews (n = 9, 12%) did not use any specific terminology to characterize their research.

#### Journals: academic fields and titles

The reviews selected for analysis were identified as coming from 20 different fields, mainly from “Medical Ethics/Ethics” (18%), “Nursing” (17%), and “Healthcare Sciences & Services/Health Policy & Services/Public, Environmental, & Occupational Health” (16%) (see Table 1). The journal that published most of the selected reviews was *Nursing Ethics* with 15 (20%); overall, 33% (n = 25) of the journals were nursing journals. However, the majority of the reviews (59%) were published in a journal that only published one such review on empirical literature in the field of bioethics (see Table 1).

**Table 1.** Journals (academic fields and titles) of the reviews.

Sorted after highest ranking; multiple responses possible			
Academic field (according to JCR SCIE//SSCI)—No. of journals (% from total n = 76)			
Medical Ethics/Ethics	14 (18%)	Multidisciplinary Science//Engineering, Multidisciplinary	2 (3%)
Nursing	13 (17%)		
Healthcare Sciences & Services/Health Policy & Services//Public, Environmental, & Occupational Health	12 (16%)	Surgery	2 (3%)
		Psychiatry	2 (3%)
		Oncology	1 (1%)
Social Issues/Social Science, Biomedical	8 (11%)	Pharmacology & Pharmacy	1 (1%)
Medicine, General & Internal/Primary Healthcare	6 (8%)	Transplantation	1 (1%)
		History & Philosophy of Science	1 (1%)
Geriatrics & Gerontology/Gerontology	5 (7%)	Nutrition & Dietetics	1 (1%)
Genetics & Heredity	5 (7%)	Critical Care Medicine	1 (1%)
Biotechnology & Applied Microbiology/Immunology	4 (5%)	Emergency Medicine	1 (1%)
	
Medicine, Research & Experimental	3 (4%)	Not found in JCR Science/Social Science Edition 2018	2 (3%)
Obstetrics & Gynecology/Reproductive Biology	3 (4%)		
Journal title—No. of reviews (% from total n = 76)			
*Nursing Ethics*	15 (20%)	*PLOS One*	2 (3%)
*Journal of Medical Ethics*	6 (8%)	*Transplantation*	2 (3%)
*Journal of Advanced Nursing*	4 (5%)		
*Health Policy*	2 (3%)	Journals with only one published article^a^	45 (59%)

JCR: journal citation reports; SCIE: science edition; SSCI: social science.

^a^ Including six nursing journals.

#### Authors: number, country of origin, and affiliations

The reviews were authored by 1 till up to 10 or more researchers; however, 70% were written by groups of 2 to 4 authors (see Table 2); 48% of the first authors were located in English-speaking countries (the United States, Canada, and the United Kingdom), 41% in other European countries (excluding United Kingdom), 3% in Brazil, 1% in South Africa, and 1% in Israel (see Table 2). A large number of the authors were affiliated with an institution that can be assigned to “Medicine” (18%), “Nursing/Allied Health Professions (AHP)” (17%) or “Bioethics” (14%) while some were affiliated with institutions from “Public Health/Global Health/International Health” (10%), “Health Science” (7%), or “Social Sciences” (7%) (see Table 2).

**Table 2.** Authors (number, country of origin, and affiliation) of the reviews.

No. of authors—No. of reviews (% from total n = 76)														
1 author	6 (8%)		3 authors		24 (31%)		5 authors		7 (9%)			7 authors		2 (3%)
2 authors	13 (17%)		4 authors		16 (21%)		6 authors		5 (7%)			>7 authors		3 (4%)
Country of origin (according to the first author)—No. of reviews (% from total n = 76)														
The United States		13 (17%)		The Netherlands			3 (4%)			France			1 (1%)	
Canada		12 (16%)		Brazil			2 (3%)			Ireland			1 (1%)	
The United Kingdom		11 (15%)		Sweden			2 (3%)			Israel			1 (1%)	
Belgium		7 (9%)		Switzerland			2 (3%)			Norway			1 (1%)	
Finland		6 (8%)		Portugal			2 (3%)			South Africa			1 (1%)	
Australia		5 (7%)		Croatia			1 (1%)			Spain			1 (1%)	
Germany		3 (4%)		Cyprus			1 (1%)							
Affiliation—No. of authors (% from total no. of authors, n = 170)														
						First author^a^ (n = 84)		Last author^b^ (n = 27)			Other authors^b^ (n = 59)			Total (n = 170)
Medicine						12		3			15			30 (18%)
Nursing/AHP						20		2			7			29 (17%)
Bioethics						17		4			4			25 (14%)
Public Health/Global Health/International Health						7		5			5			17 (10%)
Health Science						7		1			4			12 (7%)
Social Sciences (including Economics)						2		4			6			12 (7%)
IT/Communication						1		2			1			4 (2%)
Law/Politics						2		1			1			4 (2%)
Philosophy/Humanities (including Ethics in general)						3		0			1			4 (2%)
Health Insurances						0		0			3			3 (2%)
Genetics						1		0			1			2 (1%)
Statistics						0		0			1			1 (1%)
Other (unspecific)						7		2			7			16 (9%)
Not stated						5		3			3			11 (6%)

AHP: allied health professions.

^a^ Some first authors had more than one affiliation.

^b^ If different from the first author or first and last author.

#### Reviews’ main topics

We classified the various topics covered in the reviews in three main areas of applied ethics: clinical ethics (n = 38, 50%), research ethics (n = 27, 36%), and public health ethics or organizational ethics (n = 11, 14%). The issues most often addressed in the reviews are “ethics at end of life” (17%), “ethical competence of nurses” (15%), “informed consent” mainly in research contexts (10%), and “Healthcare management/organization” (10%) (see Table 3).

**Table 3.** Topics/subject areas of the reviews.

Topic—No. of reviews (% from total n = 76)						
Clinical Ethics				Research Ethics		
Ethics at the end of life		13 (17%)		Informed consent		8 (10%)^a^
Ethical competence of nurses		12 (15%)		Publication ethics		4 (5%)
Organ donation		3 (4%)		Research integrity		3 (4%)
Ethics in mental healthcare		2 (3%)		Ethics of genetic research		3 (4%)
Ethics of genetic testing		2 (3%)		Public involvement/participatory initiatives		2 (3%)
Ethics of reproductive technologies		2 (3%)		Quality of (reporting of) research		2 (3%)
Ethics in general medical practice		1 (1%)		Research on vulnerable populations		2 (3%)
Ethics education		1 (1%)		Embryos donation for research		1 (1%)
Quality of care from an ethical perspective		1 (1%)		Empirical ethics research in pharmacy		1 (1%)
Conceptualization of “everyday ethics”		1 (1%)		Characterization and evaluation of IRBs		1 (1%)
Public Health Ethics or Organizational Ethics						
Healthcare management/healthcare organization		8 (10%)		Care rationing/prioritization		3 (4%)

IRB: institutional review board.

^a^ One review synthesized studies about informed consent in research as well as in treatment context.

### Methodological characteristics

#### Type of information reviewed

About 21% of the selected studies reviewed “Attitudes toward or opinions about an ethical issue or several related issues” (see Table 4). Other information types such as “Possible (causal) factor(s) associated with a decision or with an outcome” or “Experiences regarding a given ethical issue” were each reviewed by 15% of the reviews. Further information types such as “Understanding of a concept/term” were retrieved by one or two reviews (see Table 4).

**Table 4.** Type of information retrieved by the selected reviews.

	No. of reviews (% from n = 76)
Attitudes toward ethical issues (e.g. about organ donation or authorship criteria)	16 (21%)
Factors associated with a decision or an outcome (e.g. higher methodological quality of a clinical trial correlates with better reporting of ethical issues)	12 (15%)
Experiences on a given ethical issue or context (e.g. dilemmas experienced)	11 (15%)
Use, evaluation, or outcome of specific procedures or “ethics tools” (e.g. obtaining informed consent, improving understanding, promoting ethical competence)	11 (15%)
Descriptions of particular ethical challenges or ethical issues related to the topic (e.g. use of palliative sedation for nonphysical suffering, genetic cancer risk assessment technologies)	11 (15%)
Reporting in the literature (e.g. about funding sources or obtained informed consent, or about the methods used)	11 (15%)
Self-reported or observed behavior and decision-making (e.g. plagiarism, do-not-resuscitate orders)	2 (2%)
Understanding of a concept (e.g. “everyday ethics”)	1 (1%)
Descriptions and evaluations of institutional structure or process (e.g. IRBs)	1 (1%)

The type of information summarized here refers to the *main* information retrieved in the reviews. Additional information such as, for example, the ethical theories used by the included studies of an SR,^11^ are not depicted in this table. IRB: institutional review board.

#### Ethical outcome from the reviews

We analyzed the ethical outcome of the reviews through the categories “ethical reflection” and “ethical recommendation.” For “ethical reflection,” we differentiated between “practical implications,” “normative implications,” and “theoretical implications.” “Practical implications” subsume reflections about observations (e.g. what is done on the ward or in research settings), ethically relevant consequences associated with these observations, and ethical evaluation of the situation observed (i.e. the reflection stays mainly on the level of the actual practice, of actions and omissions). We defined “normative implications” as justifications, critiques, or prioritization of principles, norms, values, and concepts. Such implications could also reflect discussions on observed transgressions of or adherences to norms or existing guidelines (i.e. the reflection stays mainly on the level of moral norms, values, and concepts). Finally, “theoretical implications” were defined as related to the development of specific theories or to criticisms or modifications of existing theories (e.g. “principlism” as a theoretical approach), including implications for bioethics as a field (e.g. which topics should be addressed more intensively in bioethics). For “ethical recommendations,” we excluded unspecific recommendations such as, for example, “there should be more consideration paid to issue x” or “x should be researched more in detail.” Instead, we retrieved data from the reviews that gave some substantial context-specific recommendations based on the results of the review.

In all, 72% (n = 55) of the reviews included some sort of ethical reflections on their results; 26 of these reflections can be described as “practical implications” (34% of all reviews), 25 (33% of all reviews) as “normative implications.” The remaining 4 (5% of all reviews) had “theoretical implications.” Of the 76 reviews, 59% (n = 45) included practice-oriented ethical recommendations in their discussions or conclusions. For instance, some authors discussed ways to improve specific processes or alternatives for specific practices (see some examples in Table 5). Overall, 20% of all reviews included ethical reflections as well as ethical recommendations, while 12% of all reviews did not draw any type of ethical outcome (recommendation or reflection).

**Table 5.** Review excerpts illustrating the different ethical outcomes.

Examples of Ethical reflection with…
* …practical implications*: “Especially in nursing, a culture focused on safety and control […], it is obvious that nurses value safety first and foremost by preferring to use restraints rather than risking that a patient may fall. For nurses and their practice, this kind of ethic comes with many risks and consequences. First, by prioritizing safety, nurses run a serious risk of reducing good care to care that satisfies the patient’s physical well-being, but ignores the patient’s psychological, social, moral and spiritual well-being, thereby denying the patient his or her total well-being […]. Second, by narrowing good care to care that focuses on physical wellbeing, nurses strengthen their professional perspective towards restraint use, making it easier for them to push aside their personal perspective. Third, when nurses repeatedly make decisions that cause their personal feelings to be pushed aside, they are more apt to experience inner conflicts. Fourth, by solving conflicts by rationalizing the use of restraints, nurses run the risk of distancing themselves from their patients, which makes it even easier for them to apply restraints […].” (Goethals et al., 2012: 1206)
…*normative implications*: “The fact that nurses use their personal and societal values instead of the codes to justify their ethical decisions has been found in other studies as well (e.g. […]). It reflects a relativistic approach to ethics. This may prove problematic in professional and in current multicultural health care contexts. Reference to the common codes for all nurses might help to alleviate this.” (Numminen et al., 2009: 391)
…*theoretical implications*: “Relational ethics, as a moral philosophy, asks us to engage in, and reflect on, the relationships of the moment. Advocacy in nursing is embedded in a relational context. Burgum’s […] discussion of relational ethics echoes nurses’ experiences with advocacy in practice. Relational ethics therefore provides a meaningful perspective from which to study the nature of advocacy in nursing practice. However, it is worth noting that consideration of the impact of relational ethics on advocacy should not exclude such concepts as rights and justice in informing nurses’ advocacy choices. Rather, I would argue that the inclusion of relational ethics in the advancement of our understanding of advocacy in nursing is an important contribution to our continued study of all theories and concepts that help clarify the role of advocacy in nursing. Further study of relational ethics as it informs advocacy in nursing is critical to the expansion of our knowledge of the connection between this postmodern ethic and advocacy.” (MacDonald, 2007: 124)
Examples of Ethical recommendations
“The most important implication for nursing research is that graduate education must include not only the skills to write an ethical proposal to an ethical review board, but also those that reflect on the implementation of the research principles during the research process and demonstrate critical reflection on ethical issues involved in the research.” (Kjellström et al., 2010: 391)
“[…] However, if, as the findings here suggest, a component of bias is operating unconsciously, then there is a limit to the usefulness of disclosure as a management strategy. Therefore, policy-makers could consider banning financial ties to reduce bias, just as Dana and Lowenstein […] suggest prohibiting gifts to physicians. […] Because the findings here suggest that researchers are concerned about the perceived risks of financial ties in research, these proposed bans could protect against even the appearance of conflict.” (Glaser/Bero, 2005: 562)
*For full bibliographic details for the reviews referenced, see Supplemental File S1*

#### References to review methodology

Sixteen of the 76 reviews (21%) gave a reference for the review methodology they actually applied or used for guidance. Half of those reviews (n = 8) referred to handbooks, while the other half (n = 8) cited published methodological approaches in single papers or book chapters (see Table 6).

**Table 6.** References cited as guidance for review methodology by 16 of the reviews.

Review methodology referenced	No. of reviews citing reference (% from n = 16)
Handbooks	
“Systematic Reviews” (Center for Reviews and Dissemination)^12^	4 (25%)
“Reviewer’s Manual” (Joanna Briggs Institute)^13^	2 (13%)
“Cochrane Handbook for Systematic Reviews of Interventions” (Cochrane Collaboration)^14^	1 (6%)
“Doing a literature review in health and social care: a practical guide” (Aveyard)^15^	1 (6%)
Published methodological approaches (in papers or as book chapters)	
“Systematic reviews of empirical bioethics”^7^	3 (19%)
“Methodologic guidelines for systematic reviews of randomized control trials in health care from the potsdam consultation on meta-analysis”^16^	1 (6%)
“Scientific guidelines for conducting integrative research reviews”^17^	1 (6%)
“Information on ethical issues in health technology assessment: how and where to find them?”^18^	1 (6%)
“Review: a narrative review of the published ethical debates in palliative care research and an assessment of their adequacy to inform research governance”^19^	1 (6%)
“Synthesis: combining results systematically and appropriately by Thomas et al.^20^	1 (6%)

#### References to quality appraisal methods/tools

Of the 76 reviews, n = 39 (51%) included a statement regarding the quality or critical appraisal of the included studies. Although they included such a statement, three of these reviews did not provide references; neither did they describe their quality appraisal process. Of the remaining reviews (n = 36), 39% referred to guidelines or tools, 44% to specific approaches published in papers or book chapters, and 22% described their own criteria or methods (see Table 7).

**Table 7.** Quality appraisal methods, guidelines, or approaches cited by 36 of the reviews.

Quality appraisal methods referenced	No. of reviews describing quality appraisal (% from n = 36) (multiple responses possible)
Guideline/tools	
CASP (Critical Appraisal Skills Program) (National Health Services, NHS)^21^	6 (17%)
COREQ (Consolidated Criteria for Reporting Qualitative Research)^22^	3 (8%)
Quality Appraisal Checklist—Qualitative Studies (National Institute for Health and Care Excellence, NICE)^23^	1 (3%)
Guidelines for Critical Review Form: Qualitative studies^24^	1 (3%)
Online standardized critical appraisal Joanna Briggs Institute Qualitative Assessment and Review Instrument (JBI-QARI)^25^	1 (3%)
STROBE (Strengthening the Reporting of Observational Studies in Epidemiology)^26^	1 (3%)
Systematic Review Study Quality Form^27^	1 (3%)
Specific approaches (in papers, handbooks)	
“Appraising the evidence: reviewing disparate data systematically”^28^	3 (8%)
“Standard Quality Assessment Criteria for Evaluating Primary Research Papers from a Variety of Fields”^29^	3 (8%)
“Conducting a critical interpretive synthesis of the literature on access to healthcare by vulnerable groups”^30^	1 (3%)
“Developing evidence based social care policy and practice. Part 3: Feasibility of undertaking systematic reviews in social care”^31^	1 (3%)
“Evaluation of empathy measurement tools in nursing: systematic review”^32^	1 (3%)
“How to read a paper: the basics of evidence based medicine”^33^	1 (3%)
“Methodologic guidelines for systematic reviews of randomized control trials in health care from the Potsdam consultation on meta-analysis”^16^	1 (3%)
“Nursing Research. Generating and Assessing Evidence For Nursing Practice”^34^	1 (3%)
“Qualitative Research and Cochrane Reviews”^35^	1 (3%)
“Reading qualitative studies”^36^	1 (3%)
“Studies of symptoms in primary care”^37^	1 (3%)
“Synthesizing qualitative and quantitative evidence: a review of possible methods”^38^	1 (3%)
* Own criteria used (without references to particular guidelines or approaches, etc.)*	8 (22%)

### Reporting quality

Of the 76 reviews, 18% referred explicitly to PRISMA for their reporting strategy (“PRISMA”-subgroup, blue pole in Figure 3) while 82% did not (“Non-PRISMA”-subgroup, red pole in Figure 3). Evaluation with the slightly adapted PRISMA checklist^4^ showed that the PRISMA subgroup reported on average more comprehensively than the other group. In four categories, the difference between the PRISMA and the Non-PRISMA subgroups is over 30 percentage points, and more than 15 percentage points in another six categories (see Figure 3). The two main differences were (1) the title (66 percentage points), which according to PRISMA should explicitly refer to a “systematic review” and (2) the statement of the date/period of the search(es) (40 percentage points). The only exception was “statement of used search restrictions” where the Non-PRISMA subgroup had 18 percentage points more than the PRISMA subgroup (see Figure 3).

Generally, the reporting criteria were met differently not only in the former subgroups but also when considering the total amount of reviews (marked with a “T” (total) in Figure 3). For example, nearly all (n = 75, 99%) state the databases they used for literature search as well as the kind of information they sought (n = 74, 97%). However few reviews stated the theoretical approaches used to define the information (n = 10, 13%) or the synthesis method they applied (n = 18, 24%) (see Figure 3). Although most reviews (96%) reported the number of hits finally included, 74% reported the number of hits initially found (see Figure 3). Regarding the limitations statements, while 74% reported limitations of their review, 55% reported general limitations linked to the literature or database, and 24% reported limitations of each study included (see Figure 3).

**Figure 4.**
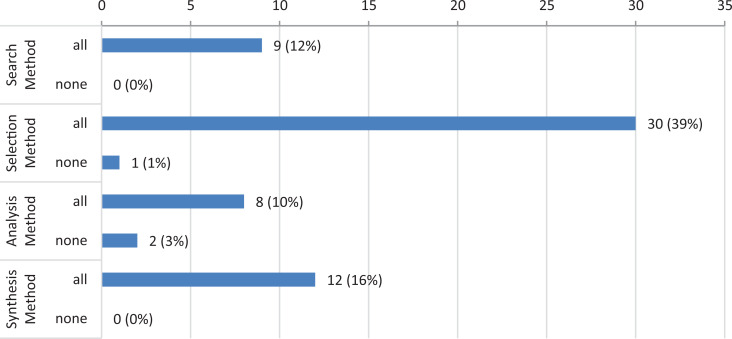
Reporting criteria of the reviews: all or no criteria met in different method categories (n = 76).

While each of the selected reviews reported some item of the PRISMA reporting criteria, three of them gave no statements on one of the four major methodological steps (search = 1; analysis = 2) (see Figure 4). Notably the reporting of the selection process fulfilled all PRISMA criteria in 39% of the reviews selected in our study (see Figure 4).

## Discussion

In our meta-review, we included 76 reviews, published between 1997 and 2017, that reviewed (i.e. searched, analyzed, and synthesized) empirical literature on specific bioethical topics. Systematic reviews of empirical literature are emerging (83% published in the last 10 years) and are represented in a variety of journals (76 reviews have been published in 51 different journals from 20 academic fields according to JCR). Nursing ethics currently plays a dominant role in this new field. Our results indicate that nursing (ethics) journals are the major publication organs (33%), first authors most prominently come from nursing departments (24%; 20 of 84 authors, see Table 2), and the topics addressed in the reviews are, in their large majority, explicit nursing issues (“ethical competence of nurses”) or closely associated with the nursing care (“ethics at the end of life”) (69% of the “clinical ethics” topics (n = 38, see table 3)).

However, the overall heterogeneity of our sample, further revealed by the variety of authors’ affiliations (n = 12), can be explained by the interdisciplinary nature of bioethics. Nevertheless, our meta-review unveiled some characteristics common to such reviews of the literature. These common features include authors’ number (groups of 2 to 4 wrote 70% of our sample) and authors’ professions (healthcare workers wrote 35% of the reviews and bioethicists or philosophers 16% of our sample). Further shared features include identifiability by the term “systematic review” or a comparable term in the title (46%); written by authors located in the United States, Canada, or the United Kingdom (48%); and topic related to a clinical-ethical issue (50%), especially the end of life (17%) and healthcare professionals’ “ethical competence” (15%). None of the information types extracted in the systematic reviews particularly stands out. However, the following six types (96%) were reviewed in similar proportions: attitudes or opinions (21%), influencing factors, experiences, instruments or tools, challenges/issues, or ethical reporting (15% each). Most of the selected reviews did not refer to established or published approaches for systematic reviews (79%) neither did they refer to reporting guidelines (82%). Still half of our sample mentioned some form of quality appraisal (51%). Most of the selected reviews included ethical reflections (71%) and more than half of them drew practice-oriented ethical recommendations from their analysis (59%).

That only about half (46%) of the selected reviews described themselves as a “systematic review” (see Figure 3) might in part depend on the acceptance of the method especially in journals of bioethics or the field of bioethics in general. However, it could also be influenced by the established perception of “systematic review” as a quantitatively oriented approach summarizing evidence related to health interventions, from which systematic reviews of empirical literature in bioethics depart. Also, in qualitative social research, there seems to be a tendency to give such literature reviews distinct names (such as “critical review,” “comparative review,” or “thematic synthesis”).^39^ Nonetheless, one could have expected more reviews of empirical literature to identify themselves as “systematic reviews” as the method originally aims at finding and synthesizing empirical quantitative studies in a structured manner.

The dominance of clinical-ethical questions in the selection of topics, on the contrary, might not be so surprising given the background and affiliations of the authors. In addition, systematic literature reviews are only meaningful when there is actual scientific work available on a certain topic. As clinical-ethical topics such as “end-of-life decision-making” are very much discussed in the literature, they present a favorable ground to perform reviews.

The fact that most of the selected reviews included ethical reflections and/or recommendations may be important to distinguish them from reviews coming from other perspectives or fields such as sociology or psychology. Indeed, the latter type of studies may also include ethically relevant results but are not conducted with the *intention* of providing knowledge for ethical analysis and/or assessment regarding the topic at hand. Against this backdrop, it is also explainable that the ethical reflections are mostly *practical implications* (34%) and *normative implications* (33%), and less *theoretical implications* (5%)—even when considering sometimes fluid boundaries between these categories. This might, in addition, be due to the empirical nature of the studies reviewed, which may influence the nature of the ethical outcome. Also, the generally more practice-oriented nature of interdisciplinary bioethics^40^ might play a role, as the ethical outcome would possibly be different if such reviews were conducted by researchers working in philosophical ethics (cf. approaches of experimental philosophy^41^ in which empirical work is mainly used to clarify philosophical questions).

We could explain the fact that few reviews referred to established review methodology (21%) or to reporting guideline (18%) in two different ways. On one hand, the body of available guidance may be small or difficult to access. On the other hand, reporting a specific methodology for conducting systematic review may not be perceived as necessary because performing systematic reviews of empirical studies has become relatively common practice in contemporary research.

There is some truth to both points. One of the key strengths of a systematic review is its explicit orientation toward a methodology that ensures reproducibility and transparency. Referring to a given methodology may help authors to limit their description to deviations from standard procedures in order to focus on their particular issues. For reviews of empirical studies on bioethical topics, these specificities may include the ethical framing of the research question, the definition of information units, a way to analyze and synthesize information in order to facilitate an ethical assessment, or the incorporation of ethical reflections in the results. While such particularities need to be reflected upon in the methodological elaborations, they have not yet been sufficiently addressed in consented methodological and reporting standards. The lack of uniformity in the methodological and reporting standards already noticed^42^ was confirmed in our meta-review in which a single reference for systematic review methodology was cited by a maximum of four reviews. So, this meta-review and the findings on systematic reviews of normative literature on bioethical topics^4,8^ can serve as an inventory, providing a sound basis for discussion of best practice and reporting standards.

Finally, looking at the quality of reporting according to some selected PRISMA statements, it is striking that flowcharts are not yet common practice (51% of the reviews) (see Figure 3)—surprisingly even when the reviews referred to PRISMA (only 58% of those 14 reviews). The search is often not completely reproducible on the basis of the reporting (e.g. statement of date/period of search (38%) or statement of used search restrictions (60%), but at least 91% described the search terms or the search string (see Figure 3)). Nonetheless, it has to be noted that at least one of the criteria of the respective method categories (search, selection, analysis, and synthesis) was met by nearly all selected studies (see Figure 4). Compared to other studies that checked reporting quality according to PRISMA, it can be said that the introduction of PRISMA in 2009 has led to the improvement of the reporting quality,^43,44^ and so does its explicit endorsement.^45–47^ The better reporting on average in the PRISMA subgroup versus the Non-PRISMA subgroups could be attributed to using the PRISMA checklist. However, we have to acknowledge that, in light of the small numbers of the subgroup analysis, this could also be a result of chance and it cannot be excluded that other causes might also have been relevant. On the whole, our results illustrate, once more, that reporting guidelines specific for reviews on bioethical topics would be useful, not only for systematic reviews of normative literature^8^ but also for systematic reviews of empirical literature.

### Systematic reviews of empirical literature versus systematic reviews of normative literature

A comparison of the results of this review on empirical literature with those of normative (including mixed) literature^4,8^ shows differences and similarities. The sample (n = 84) of systematic reviews of *normative* literature on bioethical topics was also characterized by a certain heterogeneity, which was revealed by the number of different fields (n = 38), journals (n = 65), and affiliations of the authors (n = 10). However, reviews of normative literature share the following features: two to four authors (71%), mainly healthcare professionals (medicine/nursing/AHP, 37%) or bioethicist/philosophers (32%); 37% are identifiable by the term “systematic review” in the title, and they are written by authors from the United States or United Kingdom (40%), or from Belgium, Germany, or the Netherlands (30%). A typical review applies qualitative methods as used in social science research (83%) and focus on one of the following information types: challenges/issues (33%), arguments/reasons (17%), and principles/values/norms (17%). Furthermore, only 24% refer to established or published approaches for conducting systematic reviews, 12% to reporting guidelines, and 24% inform about quality appraisal.

All systematic reviews (empirical, normative, and mixed) have in common that it is a new and emerging study type within bioethics (more than 80% published in the last 10 years) and that nursing is strongly represented both as academic field and as publishing journals. Differences (empirical vs normative and mixed) can be observed in the authors’ affiliations. There, for systematic reviews of normative literature, “Bioethics” was the leading category in total (29%), followed by “Medicine (26%) and “Nursing/AHP” (11%) (see Table 4 in Mertz et al. 2016).^4^ For systematic reviews of empirical literature, “Medicine” is leading (18%), followed by “Nursing/AHP” (17%) and “Bioethics” (14%) (see Table 2). This, however, was expectable given that systematic reviews of normative literature are close to the traditional normative work of bioethics with one of its roots in philosophy. In contrast, reviews of empirical studies seem to be closer to traditional reviews and empirical disciplines such as medicine or nursing. As a hypothesis, it might additionally be that bioethicists are more inclined to conduct systematic reviews of normative literature and researchers from other disciplines, especially empirical ones, more inclined to conduct systematic reviews of empirical literature. These observations could also explain why systematic reviews of normative literature labeled themselves less often as “systematic review” (37%) compared to those of empirical literature (45%).

For quality appraisal, only 18% of the systematic reviews of normative literature reported about quality appraisal methods used in their review—another five authors (6%) explicitly wrote that they did no quality appraisal because of the lack of specific approaches.^8^ In contrast, half of the systematic reviews of empirical literature reported quality appraisal (51%). Referring to reporting guidelines as a means for quality appraisal (such as, for example, COREQ or STROBE, see Table 7) can, however, be problematic, because the evaluation of the quality is restricted to the information that is actually reported, and does not necessarily reflect the overall study quality.

In general, the reviews of empirical literature reported their methodology more extensively than the reviews of normative literature (see Figure 3 and Figure 4 in this article, Table 6 in Mertz et al. 2016).^4^ When comparing the report quality (systematic reviews of empirical vs normative and mixed literature), two aspects are particularly striking. First, systematic reviews of empirical literature score better on technical information, like “statement of used databases” (99% vs 93%) or representation of the search and selection procedure in a flowchart (51% vs 29%). Second, information that corresponds to the ethical dimension of the review was sparsely addressed by both groups. For example, “statement of procedure of (applying the) synthesis method” (24% vs 18%) or the theoretical background identification of the information unit (13% vs 21%). It has to be noted, though, that it would not be fair to equate “shortcomings in the reporting” automatically with “unworthy review.” Indeed, external constraints such as journal policies regarding article length may lead to the omission of some methodological information in the published article. However, evaluation of the quality of a review can only be based on what is actually reported. Notwithstanding this, the findings above allow drawing two conclusions: (1) current reporting guidelines, such as PRISMA, are more likely to be used by authors reporting on systematic reviews of empirical literature and (2) current reporting guidelines, such as PRISMA, do not yet sufficiently cover specific characteristics of reviews on bioethical topics and therefore should be adapted.

### Limitations

A first limitation of this meta-review could be that it is based on the results of an initial search that primarily sought to find reviews of normative literature. Since the normative aspect of the literature could not be represented in search algorithms, the search had to be broadened to identify reviews on bioethical topics in general. The reviews so retrieved were then sorted manually into reviews of (1) normative/mixed and (2) empirical literature. The additional search we performed for the present study focused on reviews of empirical literature and, as expected, it was again necessary to search broadly and then select manually. Through this manual selection that was performed independently by two of the authors, we have actively tried to limit the potential bias in the selection process, making sure to consider thoughtfully each article retrieved for inclusion in the final analysis.

Second, and as in any study working with qualitative data, we had to sometimes paraphrase or synthesize authors’ comments for analysis purposes. We cannot deny that, in this process, we may both have missed some meaning or introduced our own interpretation of the data. The same way, in the synthesis process, we had to define our different categories and subsume the data in one of these categories. It has also to be acknowledged that there are sometimes fluid boundaries between the applied categories (e.g. the three types of ethical reflection: practical, normative, and theoretical implication). Therefore, these categorizations should be treated with some caution and might be better understood as providing “trend statements,” rather than be interpreted as exact descriptions. Furthermore, a possible resulting bias could be that we emphasized one aspect of the original findings over another one. To mitigate these possible risks, we have continuously worked as independent pairs of authors, that is, coding and synthesizing independently to then compare in order to reach a common decision. This way, we tried to make sure that our interpretation of the data was as close to the original text as possible.

Finally, it should be stressed that the conclusions of the status-quo analysis based on the reported information in reviews, for example, on “review methodology,” are only based on 16 statements (21% of the sample), which limits its significance.

## Conclusion

Systematic reviews are an emerging study type for processing empirical data about ethically relevant topics. The heterogeneity currently observed is partly due to the interdisciplinary nature of bioethics, and partly due to the emerging nature of this research in the field of bioethics. The latter could also indicate the need to develop robust methodological standards. The prominent role played by nursing in reviews of normative literature can also be confirmed for reviews on empirical literature. Discussions about best practice or minimum standards are also needed for both types of reviews in the field of bioethics. The interest we appear to see in the nursing community for systematic reviews of ethics literature should resonate in a similar interest in refining its methods and discussion standards. The lack of an adapted reporting guideline also constitutes a barrier for the further development of this research method. The awareness of the importance of reporting quality should be further strengthened, so that health professionals, policy makers, and bioethicists themselves have an optimal information basis for their results’ interpretation as well as for their future research plans. We can reasonably expect that such methodological developments would result in reviews thoroughly systematic, increasingly valid and ultimately more meaningful. In turn, such high-quality reviews would be expected to positively impact (nursing) practice, may it be through better identification of ethical issues or improved dealing with these challenges.

## Supplemental material

Supplemental Material, S1_-_Full_list_of_included_reviews_of_empirical_literature_on_bioethical_topics - Systematic reviews of empirical literature on bioethical topics: Results from a meta-review Click here for additional data file. Supplemental Material, S1_-_Full_list_of_included_reviews_of_empirical_literature_on_bioethical_topics for Systematic reviews of empirical literature on bioethical topics: Results from a meta-review by Marcel Mertz, Hélène Nobile and Hannes Kahrass in Nursing Ethics

Supplemental Material, S2_-_Extended_Method_Description - Systematic reviews of empirical literature on bioethical topics: Results from a meta-review Click here for additional data file. Supplemental Material, S2_-_Extended_Method_Description for Systematic reviews of empirical literature on bioethical topics: Results from a meta-review by Marcel Mertz, Hélène Nobile and Hannes Kahrass in Nursing Ethics

Supplemental Material, S3_-_Coding_Matrix - Systematic reviews of empirical literature on bioethical topics: Results from a meta-review Click here for additional data file. Supplemental Material, S3_-_Coding_Matrix for Systematic reviews of empirical literature on bioethical topics: Results from a meta-review by Marcel Mertz, Hélène Nobile and Hannes Kahrass in Nursing Ethics
