# Supplementary material for: Systematic reviews of empirical literature on bioethical topics: Results from a meta-review
Source: Nurs Ethics. 2020 Apr 2;27(4):960–78. doi: 10.1177/0969733020907935 (PMC7323745; doi:10.1177/0969733020907935)
Supplement: Supplemental Material, S1_-_Full_list_of_included_reviews_of_empirical_literature_on_bioethical_topics - Systematic reviews of empirical literature on bioethical topics: Results from a meta-review [file S1_-_Full_list_of_included_reviews_of_empirical_literature_on_bioethical_topics.pdf]

S1 – Full list of included reviews of empirical literature on bioethical topics

Supplemental material for "Systematic Reviews of Empirical Literature on Bioethical Topics. Results from a Meta-Review."

Year	***Title***	***Authors***	***Journal/Link***
2017	Operationalising ethical challenges in dementia research-a systematic review of current evidence	West E<br>Stuckelberger A<br>Pautex S<br>et al	*Age and Ageing* 46(4):678-687<br>www.ncbi.nlm.nih.gov/pubmed/28104596
2017	Ethical frameworks for surrogates' end-of-life planning experiences: A qualitative systematic review	Kim H<br>Deatrick JA<br>Ulrich CM	*Nursing Ethics* 24(1):46-69<br>www.ncbi.nlm.nih.gov/pubmed/27005954
2017	Ethical Hurdles in the Prioritization of Oncology Care	de Groot F<br>Capri S<br>Castanier J-C<br>et al	*Applied Health Economics and Health Policy* 15(2):119-126<br>www.ncbi.nlm.nih.gov/pmc/articles/PMC5343076/
2017	Understanding Ethical Issues of Research Participation From the Perspective of Participating Children and Adolescents: A Systematic Review	Crane S<br>Broome ME	*Worldviews on Evidence-Based Nursing* 14(3):200-209<br>www.ncbi.nlm.nih.gov/pubmed/28207982
2016	What Is Everyday Ethics? A Review and a Proposal for an Integrative Concept.	Zizzo N<br>Bell E<br>Racine E	*The Journal of Clinical Ethics* 27(2):117-128<br>www.ncbi.nlm.nih.gov/pubmed/27333062
2016	Ethical issues experienced by healthcare workers in nursing homes: Literature review	Preshaw DH<br>Brazil K<br>McLaughlin D<br>et al	*Nursing Ethics* 23(5):490-506<br>www.ncbi.nlm.nih.gov/pubmed/25870176
2016	A systematic literature review on the ethics of palliative sedation: an update (2016)	Henry B	*Current opinion in Supportive and Palliative Care* 10(3):201-207<br>www.ncbi.nlm.nih.gov/pubmed/27380220
2016	Nurses' moral experiences of assisted death: A meta-synthesis of qualitative research	Elmore J<br>Wright DK<br>Paradis M	*Nursing Ethics* 25(8):955-972<br>www.ncbi.nlm.nih.gov/pubmed/28027675
2016	What ethics for case managers? Literature review and discussion	Corvol A<br>Moutel G<br>Somme D	*Nursing Ethics* 23(7):729-742<br>www.ncbi.nlm.nih.gov/pubmed/26038377
2016	A systematic review of ethical issues in vaccine studies involving pregnant women	Beeler JA,<br>Lambach P<br>Fulton TR<br>et al	*Human Vaccines & Immunotherapeutics* 12(8):1952–1959<br>www.ncbi.nlm.nih.gov/pmc/articles/PMC4994733/
2015	The ethical issues regarding consent to clinical trials with pre-term or sick neonates: a systematic review (framework synthesis) of the empirical research	Wilman E<br>Megone C<br>Oliver S<br>et al	*Trials* 17(1):443<br>www.ncbi.nlm.nih.gov/pmc/articles/PMC5016881/
2015	Assisted dying in dementia: a systematic review of the international literature on the attitudes of health professionals, patients, carers and the public, and the factors associated with these	Tomlinson E<br>Stott J	*International Journal of Geriatric Psychiatry* 30(1):10-20<br>www.ncbi.nlm.nih.gov/pubmed/25043718
2015	Open-Identity Sperm Donation: How Does Offering Donor-Identifying Information Relate to Donor-Conceived Offspring's Wishes and Needs?	Ravelingien A<br>Provoost V<br>Pennings G	*Journal of Bioethical Inquiry* 12(3):503-509<br>www.ncbi.nlm.nih.gov/pubmed/24996630
2015	Scientists Admitting to Plagiarism: A Meta-analysis of Surveys	Pupovac V<br>Fanelli D	*Science and Engineering Ethics* 21(5):1331-1352<br>www.ncbi.nlm.nih.gov/pubmed/25352123

2015	The expectations and attitudes of patients with chronic kidney disease toward living kidney donor transplantation: a thematic synthesis of qualitative studies	Hanson CS<br>Chadban SJ<br>Chapman JR<br>et al	*Transplantation* 99(3):540-554<br>www.ncbi.nlm.nih.gov/pubmed/25463967
2014	A mixed-method systematic review: support for ethical competence of nurses	Poikkeus T<br>Numminen O<br>Suhonen R<br>et al	*Journal of Advanced Nursing* 70(2):256-271<br>www.ncbi.nlm.nih.gov/pubmed/23865484
2014	Can quality from a care ethical perspective be assessed? A review	Kuis EE<br>Hesselink G<br>Goossensen A	*Nursing Ethics* 21(7):774-793<br>www.ncbi.nlm.nih.gov/pubmed/24106258
2014	Current practice of public involvement activities in biomedical research and innovation: a systematic qualitative review	Lander J<br>Hainz T<br>Hirschberg I<br>et al	*PloS One* 9(12):e113274<br>www.ncbi.nlm.nih.gov/pubmed/25469705
2014	Ethics and Community Involvement in Syntheses Concerning American Indian, Alaska Native, or Native Hawaiian Health: A Systematic Review	Gribble MO<br>Around Him DM	*AJOB Empirical Bioethics* 5(2):1-24<br>www.ncbi.nlm.nih.gov/pubmed/25089283
2014	Evaluating clinical ethics support in mental healthcare: A systematic literature review	Hem MH<br>Pedersen R<br>Norvoll R<br>et al	*Nursing Ethics* 22(4):452-466<br>www.ncbi.nlm.nih.gov/pubmed/25091004<br>*(epub ahead of time used; print version: 2015)*
2014	Factors associated with the donation and non-donation of embryos for research: a systematic review	Samorinha C<br>Pereira M<br>Machado H<br>et al	*Human Reproduction Update* 20(5):641-655<br>www.ncbi.nlm.nih.gov/pubmed/24907125
2014	Motivations of physicians and nurses to practice voluntary euthanasia: a systematic review	Vézina-Im LA<br>Lavoie M<br>Krol P<br>et al	*BMC Palliative Care* 13(1):20<br>www.ncbi.nlm.nih.gov/pubmed/24716567
2014	Nurses' experiences of ethical preparedness for public health emergencies and healthcare disasters: a systematic review of qualitative evidence	Johnstone MJ<br>Turale S	*Nursing & Health Sciences* 16(1):67-77<br>www.ncbi.nlm.nih.gov/pubmed/24635901
2014	Teaching ethics and professionalism in plastic surgery: a systematic review	de Blacam C<br>Vercler CJ	*Annals of Plastic Surgery* 72(4):484-488<br>www.ncbi.nlm.nih.gov/pubmed/24618742
2014	The ethical dimension of nursing care rationing: A thematic synthesis of qualitative studies	Vryonides S<br>Papastavrou E<br>Charalambous A<br>et al	*Nursing Ethics* 22(8):881-900<br>www.ncbi.nlm.nih.gov/pubmed/25367000<br>*(epub ahead of time used; print version: 2015)*
2013	Academic dishonesty today, unethical practices tomorrow?	LaDuke RD	*Journal of Professional Nursing* 29(6):402-406<br>www.ncbi.nlm.nih.gov/pubmed/24267935
2013	Factors affecting the clinical use of non-invasive prenatal testing: a mixed methods systematic review	Skirton H<br>Patch C	*Prenatal Diagnosis* 33(6):532-541<br>www.ncbi.nlm.nih.gov/pubmed/23828950
2013	Improving understanding in the research informed consent process: a systematic review of 54 interventions tested in randomized control trials	Nishimura A<br>Carey J<br>Erwin P<br>et al	*BMC Medical Ethics* 14(1):28<br>www.ncbi.nlm.nih.gov/pubmed/23879694
2013	Interventions for shared decision-making about life support in the intensive care unit: a systematic review	Kryworuchko J<br>Hill E<br>Murray MA<br>et al	*Worldviews on Evidence-Based Nursing* 10(1):3-16<br>www.ncbi.nlm.nih.gov/pubmed/22490044
2013	Perceived risks around choice and decision making at end-of-life: a literature review	Wilson F<br>Gott M<br>Ingleton C	*Palliative Medicine* 27(1):38-53<br>www.ncbi.nlm.nih.gov/pubmed/21993804

2013	Physician attitudes toward advanced directives: a literature review of variables impacting on physicians attitude toward advance directives	Coleman AM	*American Journal of Hospice & Palliative Care* 30(7):696-706<br>www.ncbi.nlm.nih.gov/pubmed/23125398
2013	Public attitudes to death and dying in the UK: a review of published literature	Cox K<br>Bird L<br>Arthur A<br>et al	*BMJ Supportive & Palliative Care* 3(1):37-45<br>www.ncbi.nlm.nih.gov/pubmed/24644327
2013	Systematic review of attitudes toward donation after cardiac death among healthcare providers and the general public	Bastami S<br>Matthes O<br>Krones T<br>et al	*Critical Care Medicine* 41(3):897-905<br>www.ncbi.nlm.nih.gov/pubmed/23328261
2013	Waiver of informed consent in pediatric resuscitation research: a systematic review	Eltorki M<br>Uleryk E<br>Freedman SB	*Academic Emergency Medicine* 20(8):822-834<br>www.ncbi.nlm.nih.gov/pubmed/24033626
2012	Informed consent for record linkage: a systematic review	da Silva MEM<br>Coeli CM<br>Ventura M<br>et al	*Journal of Medical Ethics* 38(10):639-642<br>www.ncbi.nlm.nih.gov/pubmed/22403083
2012	Publishing ethics in paediatric research: A cross-cultural comparative review	Brännström I	*Nursing Ethics* 19(2):268-278<br>www.ncbi.nlm.nih.gov/pubmed/22457386
2012	Nurses' decision-making in cases of physical restraint: a synthesis of qualitative evidence	Goethals S<br>Dierckx de Casterlé B<br>Gastmans C	*Journal of Advanced Nursing* 68(6):1198-210<br>www.ncbi.nlm.nih.gov/pubmed/22211472
2012	What potential research participants want to know about research: A systematic review	Kirkby HM<br>Calvert M<br>Draper H<br>et al	*BMJ Open* 2(3).pii: e000509<br>www.ncbi.nlm.nih.gov/pubmed/22649171
2012	Whose consent matters? Controlled donation after cardiac death and premortem organ-preserving measures	Bastami S<br>Krones T<br>Biller-Andorno N	*Transplantation* 93(10):965-969<br>www.ncbi.nlm.nih.gov/pubmed/22576161
2011	A systematic review of research on the meaning, ethics and practices of authorship across scholarly disciplines	Marušić A<br>Bošnjak L<br>Jerončić A	*PLoS One* 6(9):e23477<br>www.ncbi.nlm.nih.gov/pubmed/21931600
2011	A systematic review of the empirical literature evaluating IRBs: what we know and what we still need to learn	Abbott L<br>Grady C	*Journal of Empirical Research on Human Research Ethics* 6(1):3-19<br>www.ncbi.nlm.nih.gov/pubmed/21460582
2011	Burnout in palliative care: a systematic review	Pereira SM<br>Fonseca AM<br>Carvalho AS	*Nursing Ethics* 18(3):317-326<br>www.ncbi.nlm.nih.gov/pubmed/21558108
2011	Ethics of human genetic studies in sub-saharan Africa: the case of Cameroon through a bibliometric analysis	Wonkam A<br>Kenfack MA<br>Muna WF<br>et al	*Developing World Bioethics* 11(3):120-127<br>www.ncbi.nlm.nih.gov/pubmed/21781234
2011	Inclusion and exclusion in nutrigenetics clinical research: ethical and scientific challenges	Hurlimann T<br>Stenne R<br>Menuz V<br>et al	*Journal of Nutrigenetics and Nutrigenomics* 4(6):322-343<br>www.ncbi.nlm.nih.gov/pubmed/22301706
2011	Organizational ethics: a literature review	Suhonen R<br>Stolt M<br>Virtanen H<br>et al	*Nursing Ethics* 18(3):285-303<br>www.ncbi.nlm.nih.gov/pubmed/21558106

2011	Prevalence of depression in granted and refused requests for euthanasia and assisted suicide: a systematic review	Levene I<br>Parker M	*Journal of Medical Ethics* 37(4):205-211<br>www.ncbi.nlm.nih.gov/pubmed/21278132
2010	Ethical considerations in the collection of genetic data from critically ill patients: what do published studies reveal about potential directions for empirical ethics research?	Freeman BD<br>Kennedy CR<br>Frankel H<br>et al	*The Pharmacogenomics Journal* 10(2):77-85<br>www.ncbi.nlm.nih.gov/pubmed/19997084
2010	Literature review: status and trends of research ethics in Swedish nurses' dissertations	Kjellström S<br>Fridlund B	*Nursing Ethics* 17(3):383-392<br>www.ncbi.nlm.nih.gov/pubmed/20444779
2010	Nurses' ethical reasoning and behaviour: a literature review	Goethals S<br>Gastmans C<br>Dierckx de Casterlé B	*International Journal of Nursing Studies* 47(5):635-650<br>www.ncbi.nlm.nih.gov/pubmed/20096413
2010	Research on ethics in nursing care for older people: a literature review	Suhonen R<br>Stolt M<br>Launis V<br>et al	*Nursing Ethics* 17(3):337-352<br>www.ncbi.nlm.nih.gov/pubmed/20444775
2009	Are physicians willing to ration health care? Conflicting findings in a systematic review of survey research	Strech D<br>Persad G<br>Markmann G<br>et al	*Health Policy* 90:113-124<br>www.ncbi.nlm.nih.gov/pubmed/19070396
2009	Improving the methodologic and ethical validity of best supportive care studies in oncology: lessons from a systematic review	Cherny NI<br>Abernethy AP<br>Strasser F<br>et al	*Journal of Clinical Oncology* 27(32):5476-5486<br>www.ncbi.nlm.nih.gov/pubmed/19564538
2009	Nurses’ codes of ethics in practice and education: a review of the literature	Numminen O<br>Van Der Arend A<br>Helena Leino-Kilpi H	*Scandinavian Journal of Caring Sciences* 23(2):380-394<br>onlinelibrary.wiley.com/doi/10.1111/j.1471-6712.2008.00608.x/full
2009	Nurses' perceptions of ethical issues in the care of older people	Rees J<br>King L<br>Schmitz K	*Nursing Ethics* 16(4):436-452<br>www.ncbi.nlm.nih.gov/pubmed/19528101
2008	How physicians allocate scarce resources at the bedside: A systematic review of qualitative studies	Strech D<br>Synofzik M<br>Marckmann G	*Journal of Medicine and Philosophy* 33:80-99<br>www.ncbi.nlm.nih.gov/pubmed/18420552
2008	Ethics in neonatal pain research	Axelin A<br>Salanterä S	*Nursing Ethics* 15(4):492-499<br>www.ncbi.nlm.nih.gov/pubmed/18515438
2008	Nurses' attitudes towards artificial food or fluid administration in patients with dementia and in terminally ill patients: a review of the literature	Bryon E<br>Dierckx de Casterlé B<br>Gastmans C	*Journal of Medical Ethics* 34(6):431-436<br>www.ncbi.nlm.nih.gov/pubmed/18511614
2008	Nurses' moral sensitivity and hospital ethical climate: a literature review	Schluter J<br>Winch S<br>Holzhauser K<br>et al	*Nursing Ethics* 15(3):304-321<br>www.ncbi.nlm.nih.gov/pubmed/18388166
2007	A new prescription for empirical ethics research in pharmacy: a critical review of the literature	Cooper RJ<br>Bissell P<br>Wingfield J	*Journal of Medical Ethics* 33(2):82-86<br>www.ncbi.nlm.nih.gov/pubmed/17264193
2007	Institutional ethics policies on medical end-of-life decisions: a literature review	Lemiengre J<br>Dierckx de Casterlé B<br>Van Craen K<br>et al	*Medical Education* 83(2-3):131-143<br>www.ncbi.nlm.nih.gov/pubmed/17661891
2007	Relational ethics and advocacy in nursing: literature review	MacDonald H	*Journal of Advanced Nursing* 57(2):119-126<br>www.ncbi.nlm.nih.gov/pubmed/17214748
2006	The accuracy of surrogate decision makers: a systematic review	Shalowitz DI<br>Garrett-Mayer E<br>Wendler D	*Archives of Intern Medicine* 166(5):493-497<br>www.ncbi.nlm.nih.gov/pubmed/16534034

2005	A systematic review of practice standards and research ethics in technology-based home health care intervention programs for older adults	Marziali E<br>Serafini JM<br>McCleary L	*Journal of Aging and Health* 17(6):679-696<br>www.ncbi.nlm.nih.gov/pubmed/16377767
2005	Attitudes of academic and clinical researchers toward financial ties in research: A systematic review	Glaser BE<br>Bero LA	*Science and Engineering Ethics* 11(4):553-573<br>www.ncbi.nlm.nih.gov/pubmed/16279755
2005	Attitudes towards carrier testing in minors: a systematic review	Borry P<br>Fryns FP<br>Schotsmans P<br>et al	*Genetic Counseling* 16(4):341-352<br>www.ncbi.nlm.nih.gov/pubmed/16440876
2005	Risk assessment for inherited susceptibility to cancer: a review of the psychosocial and ethical dimensions	Lee RC<br>Kmet L<br>Cook LS<br>et al	*Genetic Testing* 9(1):66-79<br>www.ncbi.nlm.nih.gov/pubmed/15857189
2005	The complexity of nurses' attitudes toward euthanasia: a review of the literature	Berghs M<br>Dierckx de Casterlé B<br>Gastmans C	*Journal of Medical Ethics* 31(8):441-446<br>www.ncbi.nlm.nih.gov/pubmed/16076966
2004	Ethical aspects in the management of the terminally ill patient in the pediatric intensive care unit	Torreão Lde A<br>Pereira CR<br>Troster E	*Revista do Hospital das Clinicas Fac Med Sao Paulo* 59(1):3-9<br>www.ncbi.nlm.nih.gov/pubmed/15029279
2004	Nursing resistance as ethical action: literature review	Peter E<br>Lunardi VL<br>Macfarlane A	*Journal of Advanced Nursing* 46(4):403-416<br>www.ncbi.nlm.nih.gov/pubmed/15117352
2003	Reporting of informed consent and ethics committee approval in genetics studies of stroke	Meschia JF<br>Merino JG	*Journal of Medical Ethics* 29(6):371-a-372<br>www.ncbi.nlm.nih.gov/pubmed/14662820
2001	Methodological quality and reporting of ethical requirements in clinical trials	Ruiz-Canela M<br>de Irala-Estevez J<br>Martínez-González MA<br>et al	*Journal of Medical Ethics* 27(3):172-176<br>www.ncbi.nlm.nih.gov/pubmed/11417024
1999	Factors related to providers' decisions for and against withholding or withdrawing nutrition and/or hydration in adult patient care	Mahoney MA<br>Riley JM<br>Fry ST<br>et al	*The Online Journal of Knowledge Synthesis for Nursing* 6:4<br>www.ncbi.nlm.nih.gov/pubmed/12870092
1998	Getting meaningful informed consent from older adults: a structured literature review of empirical research	Sugarman J<br>McCrory DC<br>Hubal RC	*Journal of the American Geriatrics Society* 46(4):517-524<br>www.ncbi.nlm.nih.gov/pubmed/9560079
1998	Informed consent for clinical trials: in search of the "best" method	Edwards S<br>Lilford RJ<br>Thornton J<br>et al	*Social Science & Medicine* 47(11):1825-1840<br>www.ncbi.nlm.nih.gov/pubmed/9877351
1997	A systematic review of empirical research into ethics in general practice	Rogers WA	*British Journal of General Practice* 47(424):733-737<br>www.ncbi.nlm.nih.gov/pubmed/9519523
