# Supplementary material for: Systematic reviews of empirical literature on bioethical topics: Results from a meta-review
Source: Nurs Ethics. 2020 Apr 2;27(4):960–78. doi: 10.1177/0969733020907935 (PMC7323745; doi:10.1177/0969733020907935)
Supplement: Supplemental Material, S2_-_Extended_Method_Description - Systematic reviews of empirical literature on bioethical topics: Results from a meta-review [file S2_-_Extended_Method_Description.pdf]

S2 – Extended Method Description

Supplemental material for "Systematic Reviews of Empirical Literature on Bioethical Topics. Results from a Meta-Review."

- Descriptions are mainly taken from ANONYMOUS and ANONYMOUS

Protocol and registration: No review protocol was published beforehand, and the review was not registered (e.g. with PROSPERO).

Eligibility criteria: If not stated otherwise, the same criteria were applied at title/abstract level and full text level.

- Publication type: Published journal articles
- Language: Title/abstract level: only articles with at least an abstract in English, German, or French Full text level: only articles in English, German, or French

Inclusion criteria:

(a) Be explicitly concerned with empirical ethical considerations of medical topics. This meant the article had to, e.g.: i.) Pose an ethical question, ii.) Determine ethical problems/challenges, iii.) Address ethical decision making or the use of ethical frameworks for decision making, iv.) Explore ethical views or reasons for/against a decision, etc., v.) Look for/produce empirical data for ethical decision making or ethical evaluation, or vi.) Examine ethical regulations or recommendations, etc.; It was not sufficient for the abstract to mention, e.g., that the results of the study indicate that there are ethical issues;
(b) Have an identifiable description of at least some methodological elements describing a reproducible literature search (e.g., search terms, databases used, or inclusion/exclusion criteria); Only mentioning that "a review was done" was not enough;
(c) Only on full text level: be a review of empirical literature

Exclusion criteria:

(a) Not be a review of study protocols or of ethics consultation documentation;
(b) Not be solely concerned with legal analysis;
(c) Not solely address "meta" topics of (systematic) reviews, e.g., methodology of literature reviews in bioethics or for ethical aspects in HTA, methodologies of empirical ethics research or discussions about (research) ethics in (medical) systematic reviews;
(d) Only on full text level: not be a review of normative literature

Information sources: The review was based on two PubMed searches (15 April 2015, 27 April 2015), with additional searches in PhilPapers (29 April 2015) and Google Scholar (30 April 2015). For PubMed, two search strings were used. The first one was composed for screening purposes, and the second one used a refined (final) search string.

Search: We have not used a language restriction for the search in order to assess the overall amount of identifiable reviews.

- PubMed (15 and 27 April 2015, June 2017):
  Publication dates: No restriction;
  Language: No restriction;

Search string (final): *(“systematic review”[Title/Abstract] OR “systematic literature review”[Title/Abstract] OR “qualitative review”[Title/Abstract] OR “literature review”[Title/Abstract] OR “argument-based”[Title/Abstract] OR “systematic survey”[Title/Abstract] OR “systematic search”[Title/Abstract] OR “literature survey”[Title/Abstract] OR “systematische Übersichtsarbeit”) AND ((((Allocat*[Title] OR euthanasia[Title] OR “assisted dying”[Title] OR “end-of-life”[Title] OR palliative[Title] OR ration*[Title] OR attitude*[Title] OR motivation*[Title] OR decision*[Title]) AND (Ethics[Title/Abstract] OR Bioethics[Title/Abstract] OR “ethical issues”[Title/Abstract] OR ethical*[Title/Abstract] OR normative[Title/Abstract] OR “ethical guidelines”[Title/Abstract])) OR (Ethics[Title/Abstract] OR Bioethics[Title/Abstract] OR “ethical issues”[Title/Abstract] OR ethical*[Title] OR normative[Title] OR “ethical guidelines”[Title]))) NOT protocol* NOT “position statement” NOT “ethical approval” NOT ethics commit*;* Explanation: The exclusion (NOT) conditions for “protocol*”, “position statement”, “ethical approval” and “ethics commit*” were integrated to increase specificity of hits without decreasing sensitivity too much.
Hits: 441

- PhilPapers (29 April 2015)
  Publication dates: Start: no date restriction; end: 15.04.2015 (i.e., all hits after that date ignored)
  Language: No restriction
  Search mode: Basic fuzzy filter
  Mandatory key words: *“systematic” AND “review”*
  Relevance key words: *“ethics”*
  Hits: 447

- Google Scholar (30 April 2015)
  Publication dates: Start: no date restriction; end: 15.04.2015 (i.e., all hits after that date ignored)
  Language: No restriction
  Search mode: Allintitle
  Display mode: Sorted by relevance; citations deactivated
  Search string: bioethics OR ethics AND (“systematic review” OR “literature review”)
  Hits: 87

Selection: For the purpose of this meta-review on a still little standardized review area we decided to apply rather sensitive and not too restrictive selection criteria. We selected all reviews that explicitly or implicitly indicated their objective to analyze and present ethics literature in a systematic manner. To be included, reviews had to be explicitly concerned with empirical information of ethical topics. It was not deemed sufficient for the results of a review to be able to be regarded as “ethically relevant.” Furthermore, reviews should have an identifiable description of at least some methodological elements describing a reproducible literature search (e.g., search terms, databases used, or inclusion/exclusion criteria). Finally, we only included reviews written in English, German, or French. Articles were selected first according to their title or abstract, and later by full text screening. The selection was initially done by one researcher, both title/abstract and full text level. Then, a second researcher checked all the selection results (inclusion and exclusion) for consistency with the selection criteria. Discrepancies were discussed and successfully overcome via consensus-seeking discussions.

Quality appraisal: Because we aimed to assess the current state of the art of reviews of empirical ethical literature, we did not exclude reviews that did not fulfill all PRISMA criteria. Depicting the state of art must also include reviews of “relatively bad” reporting quality. Also, it is possible that certain reviews demonstrate a fair reporting of analysis and synthesis of empirical information but are not able to fulfill some basic PRISMA criteria. Excluding such reviews would deprive our review of important insights about how reviews of empirical information are analyzing and synthesizing information. Nevertheless, we present slightly adapted PRISMA ratings as part of our results (Figure 3).

Data extraction:

Coding: All texts were thoroughly read in full text and the relevant information was inserted into the prepared coding matrix (see supplemental file S3). Pre-formulated decision rules and response options were used to ensure quality and inter-rater reliability. We determined the academic fields of the journals that published included reviews based on how they were classified by the Journal Citation Reports (JCR) Science Edition 2014 and JCR Social Science Edition 2014. Where no entry was available, the journal was categorized as “not found”. We further categorized the affiliation of all authors. For this purpose, we considered the affiliation of all first authors. We took the lowest identifiable organizational unit if several organizational units/levels were mentioned. If the last author had a differing affiliation, this affiliation was also considered. Finally, if additional authors of a review had further differing affiliations, these were also considered. Therefore, the amount of authors considered regarding affiliations is not equal to the total amount of authors.

Identification of codes and themes: The method of qualitative content analysis [Schreier (2012), Mayring (2010)] was employed to analyze the literature in detail, i.e., to identify and categorize the methods used for search, selection, analysis, and synthesis, and the information given about methodology (e.g., stating aims, discussing limitations, providing a flowchart). In applying this method, we used a combined deductive and inductive strategy for building up categories [Schreier (2012)]. This was done iteratively by two researchers.

Synthesis Methodology: The qualitatively analyzed content of the reviews was synthesized into descriptive statistics assessing how often the description of methods corresponded to established (and slightly adapted) criteria of the PRISMA guideline.

References:

Schreier M. *Qualitative Content Analysis In Practice*. Thousand Oaks (CA): Sage Publications, 2012.

Mayring P. *Qualitative Inhaltsanalyse: Grundlagen und Techniken*. Weinheim: Beltz. 2010

ANONYMOUS

ANONYMOUS
