# Supplementary material for: Systematic reviews of empirical literature on bioethical topics: Results from a meta-review
Source: Nurs Ethics. 2020 Apr 2;27(4):960–78. doi: 10.1177/0969733020907935 (PMC7323745; doi:10.1177/0969733020907935)
Supplement: Supplemental Material, S3_-_Coding_Matrix - Systematic reviews of empirical literature on bioethical topics: Results from a meta-review [file S3_-_Coding_Matrix.pdf]

S3 – Coding Matrix

Supplemental material for "Systematic Reviews of Empirical Literature on Bioethical Topics. Results from a Meta-Review."

First-Order and Second-Order Categories ([DED] or [IND])	*Mode of answers/ Mode of data analysis*	*Decision rules (only if proven necessary!)*
TYPE OF A SR [DED] [OPND]		
Type of literature reviewed [DED] [OPND]	a=qualitative<br>b=quantitative<br>c=mixed (qualitative + quantitative)<br>d= reviews	For classification, it is more relevant what was actually included, not what is said what should have been included; if a review is "empty", then it will be classified according its intention (this includes instances where e.g. both normative and empirical literature was searched, but no empirical literature found; such a review will be classified as "mixed")
textual evidence / example	open; na	
Subject area	open	Concrete, close to text (keywords when it works)
SEARCH METHOD [DED] [OPND]		
Statement of used databases [IND] [CLSD]	1; 2	
If YES (1): Databases used/mentionned [DED] OPND]	open; na	
Statement of used search terms/strings [IND] [CLSD]	1; 2	
If YES (1): What is reported? [DED/IND] [CLSD]	a= search terms<br>b=search strings	
textual evidence / example	open; na	
If YES (1): Strategies/techniques/procedures/rationals used for finding/identifying search terms or building search strings? [IND] [OPND]	open; na	Rather technical comments about the strategy (not so much about the content of the research)
textual evidence / example	open; na	
If YES (1): Do the search strings look "copy-pastable"? [IND] [CLSD]	1; 2; na	
textual evidence / example	open; na	
Statement of date/period of the search(es) [IND] [CLSD]	1; 2	Hint: The difference between search date and cut-off is sometimes not clear!
textual evidence / example	open; na	

Use of search restrictions (e.g. publication dates, languages, type of literature [e.g. peer-reviewed arcticles, monographs, textbooks, gray literature ...]) [IND] [CLSD]	1; 2	Only "yes" if the restrictions are part of filter etc. used in the search software solution (e.g. database), i.e. the amount of hits is reduced technically by search algorithm. If researchers are manually including/excluding search results, restrictions fall under inclusion/exclusion criteria. If not clear enough in text, restrictions are generally subsumed under the categories "Statement of inclusion criteria" and/or "Statement of exclusion criteria"
If YES (1): Kind of search restrictions [DED/IND] [OPND]	open; na	
textual evidence / example	open; na	
Statement of additional search strategies used [IND] [CLSD]	1; 2	
If YES (1): Strategies used for additional searches [DED/IND] [OPND]	open; na	
textual evidence / example	open; na	
Statement about number of hits FOUND [DED] [CLSD]	1; 2	
If YES (1), total number of hits?	open; na	Provide exact number
If YES (1), n° of hits detailed by database?	1; 2; na	
If YES (1): n° of hits for each database?	open; na	Provide exact number
If YES (1): statement about hits found through additional search procedures?	1; 2; na	
If YES (1), n° of hits found through additional search procedures	open; na	Provide exact number
SELECTION METHOD [DED] [OPND]		
Statement of the selection procedure [IND] [CLSD]	1; 2	
If YES (1): Which selection procedure? [DED] [OPND]	open; na	
textual evidence / example	open; na	
If YES (1): Statement about different procedure used on title/abstract level and on the fulltext level [DED/IND] [OPND]	1; 2; na	
textual evidence / example	open; na	
Statement of inclusion criteria (e.g. languages, type of literature [e.g. peer-reviewed articles, monographs, textbooks, grey literature ...], relevance, setting, perspective ...) [IND] [CLSD]	1; 2	"1" only if the restrictions are NOT part of filter etc. used in the search software solution (e.g. database), i.e. the amount of hits is altered manually by researchers, not by search algorithm. When in doubt, restrictions are subsumed under this category or under "Statement of exclusion criteria". (See decision rule in category "Statement of search restrictions").
If YES (1): which inclusion criteria were used? [DED/IND] [CLSD]	open; na	
textual evidence / example		
If YES (1): Are the inclusion criteria different on title/abstract level and on the fulltext level? [DED/IND] [CLSD]	1; 2; na	

textual evidence / example	open; na	
Statement of exclusion criteria (e.g. languages, type of literature [e.g. peer-reviewed articles, monographs, textbooks, gray literature ...], relevance, setting, perspective ...) [IND] [CLSD]	1; 2	Only "yes" if the restrictions are NOT part of filter etc. used in the search software solution (e.g. database), i.e. the amount of hits is altered manually by researchers, not by search algorithm. When in doubt, restrictions are subsumed under this category or under "Statement of inclusion criteria". (See decision rule in category "Statement of search restrictions").
If YES (1): which exclusion criteria were used? [DED/IND] [CLSD]	open; na	
textual evidence / example	open; na	
If YES (1): Are the exclusion criteria different on title/abstract level and on the fulltext level? [DED/IND] [CLSD]	1; 2; na	
textual evidence / example	open; na	
Statement about the number of hits INCLUDED (excl. duplicate hits, if applicable) [DED] [CLSD]	1; 2	
If YES (1), total number of hits included?	open; na	Provide exact number
If YES (1), n° hits included detailed by database?	1; 2; na	
If YES (1): n° of hits included for each database?	open; na	Provide exact number
If YES (1): statement about hits included that were found through additional search procedures?	1; 2; na	
If YES (1), n° of hits included that were found through additional search procedures?	open; na	Provide exact number; check consistency with other numbers above…
ANALYSIS METHOD [DED] [OPND] (Analysis = data extraction at the level of individual literature, e.g. "what is the attitude of group x toward problem y?")		
Information about the data unit to be extracted in the analysis (e.g. attitudes …) [IND] [CLSD]	1; 2	
if YES (1): Kind of data unit/definition of data extraction? [DED/IND] [OPND]	open; na	Also acceptable if it is mentioned in the title of the review
textual evidence / example	open; na	
If YES (1): Use of a theoretical approach? [DED] [CLSD]	1; 2; na	Theory behind the construct of data unit; may be sociological, ethical or other
if YES (1): Which theoretical approach? [DED] [OPND]	open; na	
textual evidence / example	open; na	
Statement of procedure of data extraction? [DED] [CLSD]	1; 2	Does encompass e.g. tool for extraction (standardized sheet), but also the number of authors involved in the process (and their function/role)
if YES (1): Kind of procedure? [DED] [OPND]	open; na	
textual evidence / example	open; na	

QUALITY APPRAISAL		
Statement of quality appraisal [IND] [CSLD]	1; 2	It is sufficient if authors are addressing the topic of quality appraisal in SRs, even if they do not appraise for quality in their own SR (though a reason for this should be included, see according subcategory).
If YES (1): Quality appraisal method used? [DED/IND] [OPND]	open; na	
textual evidence / example	open; na	
If YES (1) and no quality appraisal method used: Rationale for not using quality appraisal method? [DED] [OPND]	open; na	
If YES (1), and review of mixed studies: Different appraisal methods used OR consideration of the specificities of the methodologies? [DED] [CSLD]	1; 2; na	
Statement of general limitations of the studies included? [IND] [CLSD]	1; 2	
textual evidence / example	open; na	
if YES (1): Statement of the specific limitations of each study included? [IND] [CLSD]	1; 2; na	
textual evidence / example		
SYNTHESIS METHOD [DED] [OPND] (Synthesis = merging information from data extraction, presentation of (merged) information)		
Statement of found/included study/paper characteristics [IND] [CLSD]	1; 2	
if YES (1): Kind of study/paper characteristics analysed? [DED/IND] [OPND]	open; na	Also if these characteristics are not stated for each single article
textual evidence / example	open; na	
Statement of publication trends (e.g. topics) in the literature found [IND] [CLSD]	1; 2	The analysis of the publication trends has to be performed and be described as such in order to be considered as a "yes"
If YES (1): Kind of publication trends analysed? [DED/IND] [OPND]	open; na	
textual evidence / example	open; na	
Statement of distribution and/or citing rate of topics/terms/arguments in the literature [IND] [CLSD]	1; 2	Only topic etc. and NOT methodological considerations
if YES (1): Kind of topics/terms analysed? [DED/IND] [OPND]	open; na	
textual evidence / example	open; na	
Statement/description of a synthesis method (e.g. grounded theory, qualitative content analysis, descriptive statistics …) [IND] [CLSD]	1; 2	Restrict "content analysis" to those reviews that EXCPLICITLY refer to (qualitative) content analysis ("QCA") as a method; "thematic analysis" reserverd for all approaches that look more or less as if "content analysis", but are not explicitly refered to in this manner
If YES (1), Kind of synthesis methods used for qualitative research? [IND] [OPND]	open; na	

textual evidence / example	open; na	
If YES (1), Kind of synthesis methods used for quantitative research? [IND] [OPND]	open; na	
textual evidence / example	open; na	
Statement about procedure of (applying) the synthesis method (e.g. one or two persons, dialogical processes …) [DED] [CLSD]	1; 2	
If YES (1): Kind of procedure? [DED] [OPND]	open; na	
textual evidence / example	open; na	
If YES (1): Rationale for this kind of procedure? [DED] [OPND]	open; na	
Statement of used statistical, content analysis, database etc. software (e.g. SPSS, MaXQDA, Access, Excel …) [DED] [CLSD]	1; 2	
textual evidence / example	open; na	
Illustration/Representation of a synthesis result [IND] [CLSD]	1; 2	
If YES (1): Kind of synthesis result representation? [DED/IND] [OPND]	open; na	It is not necessary that search result representation is distinct from contentual result representation; no further criteria is needed for "yes" than the fact that somewhere, what has been found is described/displayed / we also consider that a passage from an abstract should be considered here, as long as the reader has a synthetic overview of the review.
Formulation of (practice) ethical recommendations [IND] [CLSD]	1; 2	General statements ("One should do more") are not considered recommendations; recommendations have to be specific enough for directing/orientating action/behaviour, not just state goals
textual evidence / example	open;na	
Ethical reflections on (some) results [IND] [CLSD]	1; 2	
textual evidence / example	open;na	
ISSUES OF SPECIAL INTEREST [DED] [CLSD]		
Representation of search / selection procedure [DED] [CLSD]	1;2	
If YES (1): Kind of representation used? [IND] [OPND]	open;na	e.g. flowchart, table, narrative…
If YES (1): Use of the possibility to provide online material? [IND] [CLSD]		
Statement of theoretical foundation: Ethics [DED] [CLSD]	1; 2; 3	1=yes; 2=no; 3=implicit
If YES (1) or implicit (3): Kind of theoretical foundation? [DED/IND] [OPND]	open; na	
textual evidence / example	open; na	
Statement about references used for SR Methodology [DED] [CLSD]	1; 2	A reference to a previous publication is also considered relevant for this category

If YES (1): Kind of reference used? [DED] [OPND]	open; na	
textual evidence / example	open; na	
Statement of reporting standards and guidelines [DED] [CLSD]	1;2	
If YES (1): Kind of standards/guidelines (PRISMA/STROBE/CONSORT/Other)? [DED/IND] [OPND]	open; na	
Statement of limitations of the review [DED] [CLSD]	1; 2	
textual evidence / example	open; na	
If YES (1): Limitations specific for the ethical analysis? [IND] [OPND]	1; 2; na	
textual evidence / example	open; na	
If YES (1): Recommendations for overcoming limitations? [IND] [OPND]	open; na	
textual evidence / example		
DATA FOR DESCRIPTIVE PURPOSES		
Journal in which review is published in [DED] [OPND]	open	
Number of authors [DED] [OPND]	open	
Authors' affiliations [DED] [OPND]	open	
Country of origin [DED] [OPND]	open	
Comments from the researcher (not mandatory!)	open	
